# Detection and Functional Verification of Noncanonical Splice Site Mutations in Hereditary Deafness

**DOI:** 10.3389/fgene.2021.773922

**Published:** 2021-12-08

**Authors:** Penghui Chen, Longhao Wang, Yongchuan Chai, Hao Wu, Tao Yang

**Affiliations:** ^1^ Department of Otolaryngology—Head and Neck Surgery, Shanghai Ninth People’s Hospital, Shanghai Jiaotong University School of Medicine, Shanghai, China; ^2^ Ear Institute, Shanghai Jiaotong University School of Medicine, Shanghai, China; ^3^ Shanghai Key Laboratory of Translational Medicine on Ear and Nose Diseases, Shanghai, China; ^4^ Department of Otorhinolaryngology—Head and Neck Surgery, Xinhua Hospital, Shanghai Jiaotong University School of Medicine, Shanghai, China

**Keywords:** splice site mutation, RNA splicing, minigene, hereditary deafness, next-generation sequencing

## Abstract

Splice site mutations contribute to a significant portion of the genetic causes for mendelian disorders including deafness. By next-generation sequencing of 4 multiplex, autosomal dominant families and 2 simplex, autosomal recessive families with hereditary deafness, we identified a variety of candidate pathogenic variants in noncanonical splice sites of known deafness genes, which include c.1616+3A > T and c.580G > A in *EYA4*, c.322-57_322-8del in *PAX3*, c.991-15_991-13del in *DFNA5*, c.6087-3T > G in *PTPRQ* and c.164+5G > A in *USH1G*. All six variants were predicted to affect the RNA splicing by at least one of the computational tools Human Splicing Finder, NNSPLICE and NetGene2. Phenotypic segregation of the variants was confirmed in all families and is consistent with previously reported genotype-phenotype correlations of the corresponding genes. Minigene analysis showed that those splicing site variants likely have various negative impact including exon-skipping (c.1616+3A > T and c.580G > A in *EYA4*, c.991-15_991-13del in *DFNA5*), intron retention (c.322-57_322-8del in *PAX3*), exon skipping and intron retention (c.6087-3T > G in *PTPRQ*) and shortening of exon (c.164+5G > A in *USH1G*). Our study showed that the cryptic, noncanonical splice site mutations may play an important role in the molecular etiology of hereditary deafness, whose diagnosis can be facilitated by modified filtering criteria for the next-generation sequencing data, functional verification, as well as segregation, bioinformatics, and genotype-phenotype correlation analysis.

## Introduction

RNA splicing refers to the process of removing introns from the initial transcript (preRNA), transcribed from its DNA template, and connecting exons to form a continuous RNA molecule. In eukaryotic cells, sequences near the splicing sites of preRNA are conserved, which include the GT bases at the 5′ donor site of the intron, the AG bases at the 3′ acceptor site, the branch point composed of the polypyrimidine trace and splicing regulatory sequences such as exonic splicing enhancer (ESE), exonic splicing silencer (ESS), intronic splicing enhancer (ISE) and intronic splicing silencer (ISS) (Glisovic et al., 2008; Anna and Monika, 2018). The existence of these conserved sequences ensures the accurate RNA splicing, while mutations in these sequences may lead to structural alteration of the protein products and a variety of genetic disorders (Wang et al., 2012).

According to the human gene mutation database (HGMD), approximately 9% of the pathogenic mutations are splice site mutations (http://www.hgmd.cf.ac.uk/ac/index.php). The splice site mutations can be divided into four categories based on their locations: (I) canonical splice site (CSS) mutations at the intronic +1 and +2 positions of the 5′donor splicing site and the -2 and -1 positions of the 3′ receptor splicing site; (II) mutations in the junction regions from exonic 3bp to intronic 6bp and from intronic 12bp to exonic 2bp, excluding the classic region; (III) exonic missense or synonymous mutations in the ESE, ESS and UTR region; (IV) mutations in deep intron such as the branch point, ISE and ISS (Cartegni et al., 2002). The type II, III, and IV mutations were referred to as noncanonical splice site (NCSS) mutations. Like CSS mutations, NCSS mutations can abolish an existing donor or receptor splicing site, often resulting in loss of a whole exon (exon skipping). It can also indirectly activate a hidden splice site in exons or introns, sometimes resulting partial intron retention or exon shortening (Booth et al., 2018). In some cases, intronic or exonic mutations may create a new splice site, resulting in partial intron retention, exon shortening or formation of pseudo exons (Vaz-Drago et al., 2017).

Detection of NCSS mutations can be difficult, because the targeted sequences are not rigidly conserved as the canonical ones and the locations of mutations are far more variable (Anna and Monika, 2018). To this end, bioinformatic analyzing tools, such as Human Splicing Finder (HSF), Splice Site Prediction by Neural Network (NNSPLICE) and NetGene2, have been developed to evaluate the possible pathogenic effect of the splice site mutations (Anna and Monika, 2018). The results of the computational analysis, however, are only predictive. For NCSS mutations, functional studies such as *in vivo* RNA sequencing and *in vitro* minigene analysis, are often needed to verify their exact effect on RNA splicing (Anna and Monika, 2018).

Hereditary deafness has been known for its tremendous genetic heterogeneity (Kremer, 2019; Sheffield and Smith, 2019). Facilitated by recent development and widespread implication of next-generation sequencing (NGS), vast amounts of pathogenic variants in over 100 deafness-causative genes have been documented in recent years (https://hereditaryhearingloss.org/). Variants at NCSS, however, are often classified as variants of unknown significance (VUS) due to limited research methods to readily distinguish the pathogenic ones from the large number of benign polymorphisms (Wang et al., 2012). To this day, NCSS mutations have been reported and functionally verified in only a handful of deafness-causative genes, including a c.1282-12T > A in EYA4 lead to non-syndromic deafness DFNA10 (Hildebrand et al., 2007), a c.6050-15G > A mutation in CDH23 lead to atypical USH1 syndrome (Valero et al., 2019), and a number of NCSS mutations involving splicing of exon 8 in DFNA5 (Booth et al., 2018).

In this study, we identified 6 NCSS and 1 CSS mutations in *EYA4*, *PAX3*, *DFNA5*, *PTPRQ* and *USH1G* from 4 multiplex, autosomal dominant families and 2 simplex, autosomal recessive families with hereditary deafness. A workflow has been proposed for analyzing the NCSS variants in genetic hearing loss, including NGS with modified filtering criteria for NCSS mutation, familial segregation and genotype-phenotype correlation analysis, bioinformatic prediction and functional verification.

## Materials and Methods

### Subjects

**FIGURE 1:**
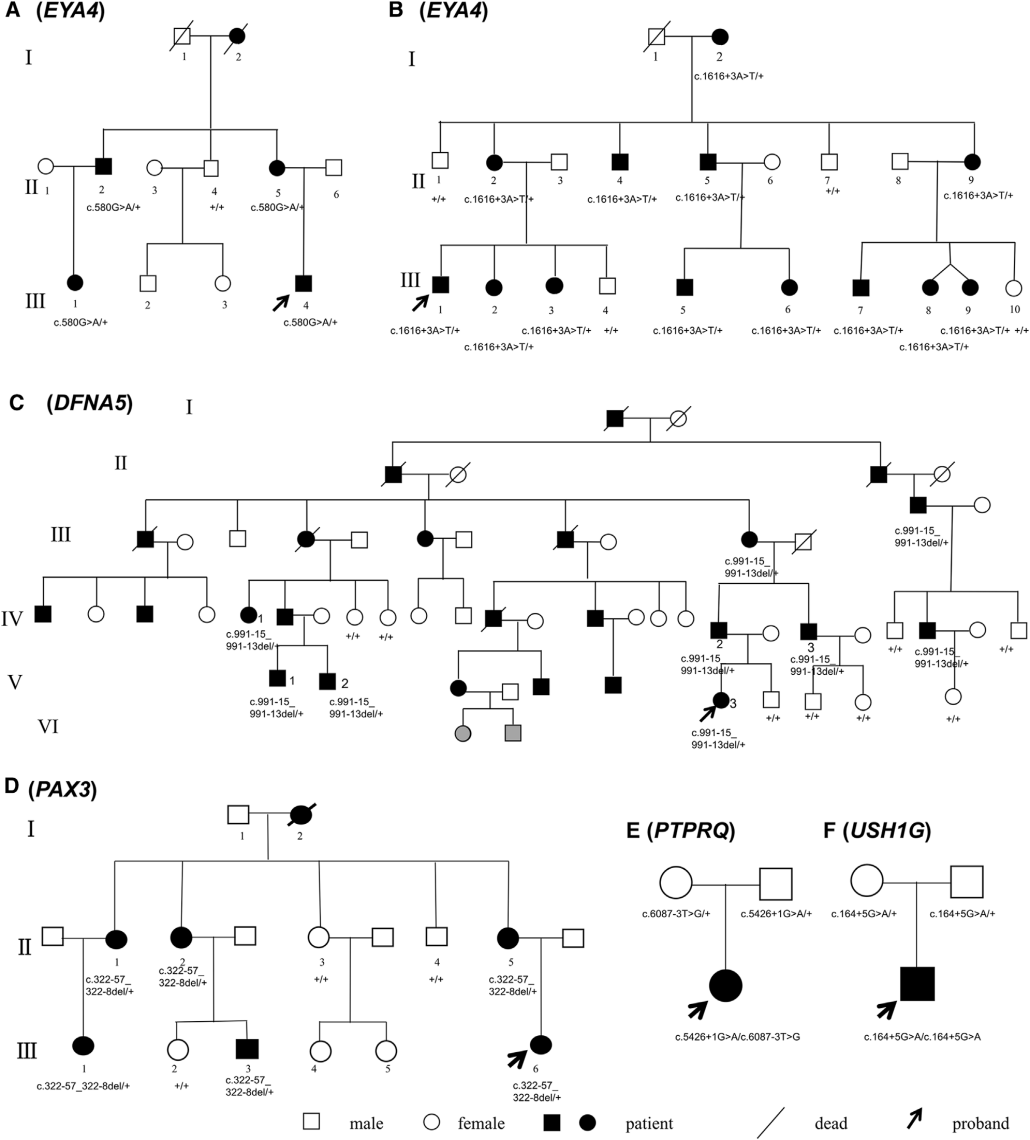
Pedigrees of Families **(A–F)**. Mutations of the causative genes (in parentheses) are marked under the corresponding individuals. Symbols in gray indicate phenotype too young to be determined.

Probands and participating members of the 4 multiplex, autosomal dominant families and 2 simplex, autosomal recessive families were recruited through the Department of Otolaryngology—Head and neck surgery, Ninth People’s Hospital, Shanghai Jiaotong University School of medicine, Shanghai, China. The pedigrees are shown in Figure 1. All subjects gave signed, informed consent to participate this study, which was approved by the ethics committee of Ninth People’s Hospital, Shanghai Jiaotong University School of Medicine.

### Next-Generation Sequencing

Genomic DNA samples were extracted from venous blood. The exons and flanking introns of 144 deafness-causative genes (Supplementary Table S1) were captured using the MyGenotics gene enrichment system (MyGenotics, Boston, MD, United States) and sequenced using the Illumina HiSeq 2000 sequencer (Illumina, San Diego, CA, United States) as previously described (Chen et al., 2016). The human genome GRCh37/hg19 was used for sequence alignment. Conventional filtering criteria were applied in the initial round of sequencing data analysis, in which only nonsynonymous variants in the coding region and the CSS variants were interrogated. In the second round of analysis, candidate variants were expanded to all intronic variants within 50 nucleotide bases from the exon boundary and synonymous/nonsynonymous variants in the exons. The minor allele frequency (MAF) for the candidate variants was set as 0.005 or less for recessive inheritance and 0.0005 or less for dominant inheritance (Shearer et al., 2014), based on public databases ESP (http://evs.gs.washington.edu/EVS/), GnomAD (https://gnomad.broadinstitute.org/) and an in-house Chinese Han population database. The pathogenicity of candidate variants was predicted by computational tools MutationTaster, PROVEAN, SIFT and Polyphen-2. Intrafamilial segregation of the candidate variants were confirmed by Sanger sequencing of all participating family members.

### Bioinformatics Analysis

Three bioinformatic analyzing tools for splicing site variants were used in this study, including HSF version 3.1 (http://www.umd.be/HSF3/HSF.shtml), NNSPLICE version 0.9 (https://www.fruitfly.org/seq_tools/splice.html) and NetGene2 (http://www.cbs.dtu.dk/services/NetGene2/).

### Minigene Analysis

The *in vitro* minigene analysis was performed as previously described (Booth et al., 2018). Wild-type and mutant minigene inserts were directly synthesized (Sangon Biotech, Shanghai, China, Supplementary Table S2) or amplified from the patients’ genomic DNA (Supplementary Table S3). The inserts were cloned into the pre-constructed exon-trap vectors pET01 (MoBiTec, Goettingen, Germany), pEGFP-C1 or pcMINI (Wuhan bioegle Biological Technology and Science Co., Ltd., Wuhan, China), all with intrinsic 3′ and 5′ exons separated by a multiple cloning site (MCS). The minigene constructs were then transfected into COS7 cells (ATCC_CRL1651)using LipofectamineTM3000 Transfection Reagent (Thermo Fisher Scientific, Waltham, Massachusetts, United States). Cells were harvested 36 h after transfection. The total RNA was extracted using the Trizol method. cDNA was reversely transcribed by TaqMan Reverse Transcription Reagents (Takara Bio Inc., Japan). The spliced products were PCR amplified with intrinsic primers from the pET01, pEGFP-C1 or pcMINI vectors, detected by agarose gel electrophoresis and Sanger sequencing.

## Results

### Clinical Characteristics of the Families With Deafness

**FIGURE 2:**
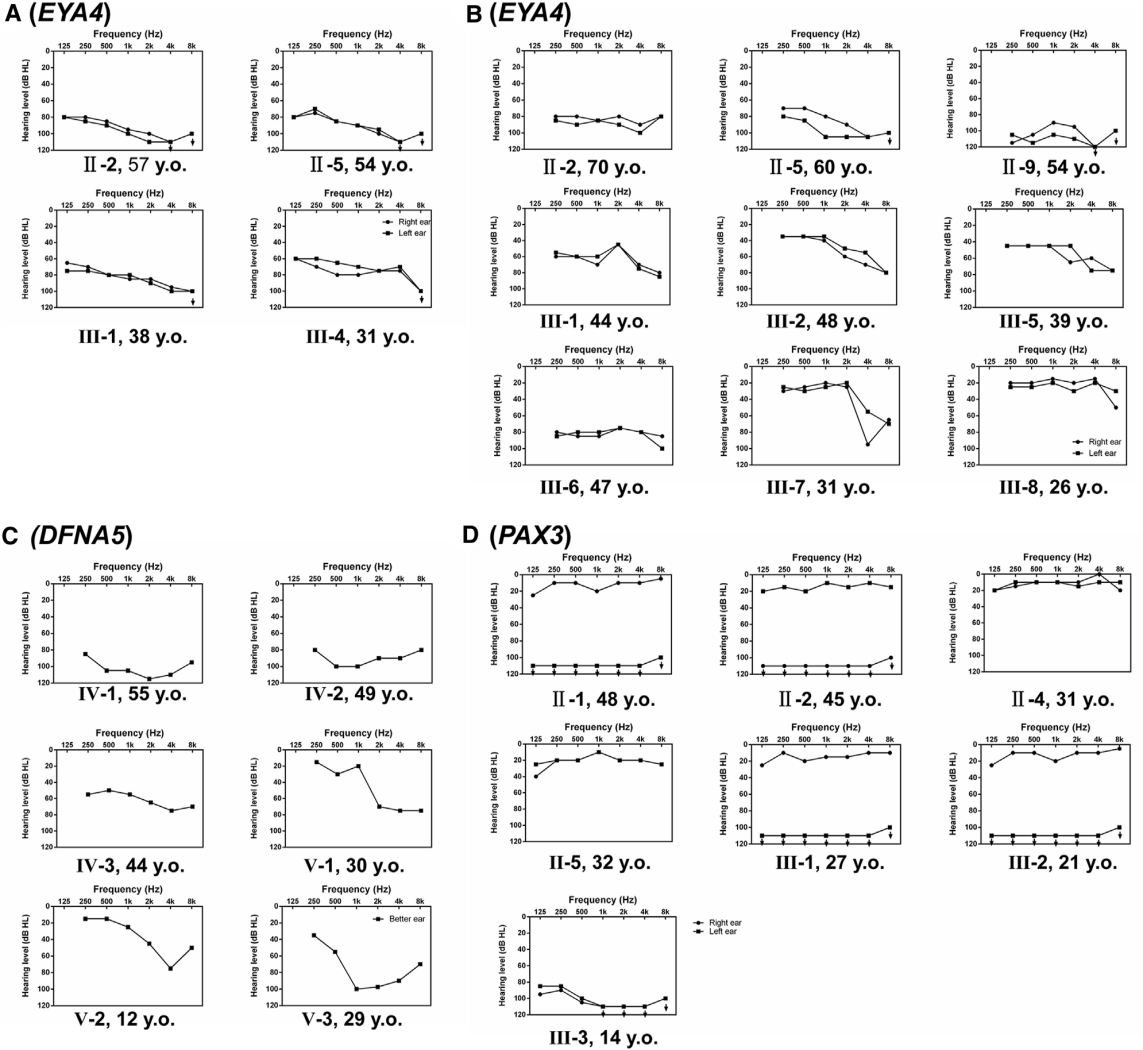
Audiograms of the affected individuals in Families **(A–D)**, which shows hearing of both ears **(A,B,D)** or the better ear **(C)**.

Families A-D exhibited typical autosomal dominant inheritance with multiple affected members spanning at least three generations (Figure 1). For Families A, B and C, audiograms of the affected members were consistent within each family, all showing bilateral, symmetric, progressive sensorineural hearing loss with delayed onsets (Figure 2). No other symptoms were reported. The affected members in Family D show characteristic features of Waardenburg syndrome type III (WS3) including sensorineural deafness, heterochromic iridis, premature graying of the hair, dystopia canthorum, patchy de-pigmentation of the skin, dystrophia canthorum and upper limb anomalies with intrafamilial variation (Table 1). Interestingly, four affected members in Family D exhibited unilateral hearing loss, which is extremely rare in genetic hearing loss. In the two simplex, autosomal recessive families (Figures 1E,F), both affected children had non-syndromic, congenital, profound deafness.

**TABLE 1:** Characteristic features of WS3 in Family D.

Status	Member	Deafness	Heterochromic iridis	Premature graying of the hair	Dystopia canthorum	Patchy de-pigmentation of the skin	Limb anomaly
Affected	II-1	Left ear	N	N	Y	N	N
Affected	II-2	Right ear	Both	Y	Y	N	N
Unaffected	II-3	N	N	N	N	N	N
Unaffected	II-4	N	N	N	N	N	N
Affected	II-5	N	N	N	Y	N	N
Affected	III-1	Left ear	Right eye	Y	Y	N	N
Unaffected	III-2	N	N	N	N	N	N
Affected	III-3	Both	Right eye	N	Y	N	N
Unaffected	III-4	N	N	N	N	N	N
Unaffected	III-5	N	N	N	N	N	N
Affected	III-6	Both	Both	N	Y	Y	Y

### Identification and Verification of the Candidate Pathogenic Variants

By targeted NGS, we identified candidate pathogenic variants in each of the multiplex, autosomal dominant families, including c.580G > A in *EYA4* for Family A, c.1616+3A > T in *EYA4* for Family B, c.991-15_991-13del in *DFNA5* for Family C, and c.322-57_322-8del in *PAX3* for Family D. Co-segregation of the variants and the disease phenotype were confirmed in all participating family members (Figure 1). The audiograms and associating features (the vestibular disorder) in Family C and the WS3-associated phenotype in Family D are consistent with previously reported genotype-phenotype correlation for the corresponding genes (Somashekar et al., 2020). In the two simplex families, compound heterozygous variants c.5426+1G > A and c.6087-3T > G in *PTPRQ* were identified in the proband of Family E and homozygous variant c.164+5G > A in *USH1G* in Family F. No other candidate variants in known deafness genes have been identified.

### Bioinformatic Analysis for the Pathogenicity of the Candidate Variants

Results of the bioinformatic analysis were shown in Table 2. Because of the close vicinity of the *EYA4* c.580G > A variant right next to the donor splice site, here we evaluated its potential negative effect on splicing instead of the presumed amino acid substitution (p.Asp194Asn). Beside the canonical c.5426+1G > A of *PTPRQ* variant, the rest of the six variants identified in this study were all type II (c.580G > A of *EYA4*, c.1616+3G > A of *EYA4*, c.6087-3T > G of *PTPRQ* and c.164+5G > A of *USH1G*) or type IV (c.991-15_991-13del of *DFNA5* and c.322-57_322-8del of *PAX3*) non-canonical splice site mutations. For the non-canonical splice site variants, HSF predicts that c.6087-3T > G of *PTPRQ*, c.991-15_991-13del of *DFNA5* and c.322-57_322-8del of *PAX3* create new acceptor sites, while c.580G > A of *EYA4*, c.1616+3G > A of *EYA4* and c.164+5G > A of *USH1G* disrupt the original splice sites. NetGene2 and NNSPLICE predict that c.1616+3G > A of *EYA4*, c.322-57_322-8del of *PAX3,* c.6087-3T > G of *PTPRQ* and c.164+5G > A of *USH1G* (weakly) disrupted the original splice sites. None of the variants were present in public databases ESP (http://evs.gs.washington.edu/EVS/), GnomAD (https://gnomad.broadinstitute.org/) and the in-house Chinese Han population database of 1,000 individuals.

**TABLE 2:** Summary and bioinformatic analysis of the pathogenic variants identified in Families A-F.

Family	Gene	Reference transcription	Candidate variants	The types of splice site mutations	HSF^a^	NetGene2^a^	NNSPLICE^a^	MAF	ACMG classification	Expression in blood
A	*EYA4*	NM_172103	c.580G > A	II (junctional)	Disruption of the original donor splice site (0.78 > 0.67)	Cannot be predicted	Cannot be predicted	0	Uncertain	extremely low
B	*EYA4*	NM_172103	c.1616+3G > A	II (junctional)	Disruption of the original donor splice site (0.10 > 0.06)	weak change of the original splice site (0.47 > 0.41)	weak change of the original splice site (1.00 > 0.99)	0	Uncertain	extremely low
C	*DFNA5*	NM_004403	c.991-15_991-13del	IV (deep intronic)	Creation of a new acceptor site (0.52 > 0.88)	No change of the original splice site (1.00 > 1.00)	No change of the original splice site (0.93 > 0.94)	0	Uncertain	extremely low
D	*PAX3*	NM_181459	c.322-57_322-8del	IV (deep intronic)	Creation of a new acceptor site (0.30 > 0.84)	Disruption of the original splice site (0.43 > 0)	Disruption of the original splice site (0.52 > 0)	0	Uncertain	extremely low
E	*PTPRQ*	NM_001145026	c.5426+1G > A	I (canonical)	Disruption of the original splice site (0.89 > 0.63)	Disruption of the original splice site (0.47 > 0)	Disruption of the original splice site (0.99 > 0)	0	likely pathogenic	extremely low
E	*PTPRQ*	NM_001145026	c.6087-3T > G	II (junctional)	Creation of a new acceptor site (0.61 > 0.66)	Disruption of the original splice site (0.26 > 0)	Disruption of the original splice site (0.93 > 0)	0	Uncertain	extremely low
F	*USH1G*	NM_173477	c.164+5G > A	II (junctional)	Disruption of the original splice site (0.91 > 0.81)	Disruption of the original splice site (1.00 > 0.71)	Disruption of the original splice site (0.99 > 0)	0	Uncertain	extremely low

a The numbers in the parentheses indicate the confidence scores of a newly created or original splicing site before and after mutation, which range from 0 (strongly disruptive) to 1 (strongly supportive).

### Verification of Disrupted Splicing for the NCSS Variants

**FIGURE 3:**
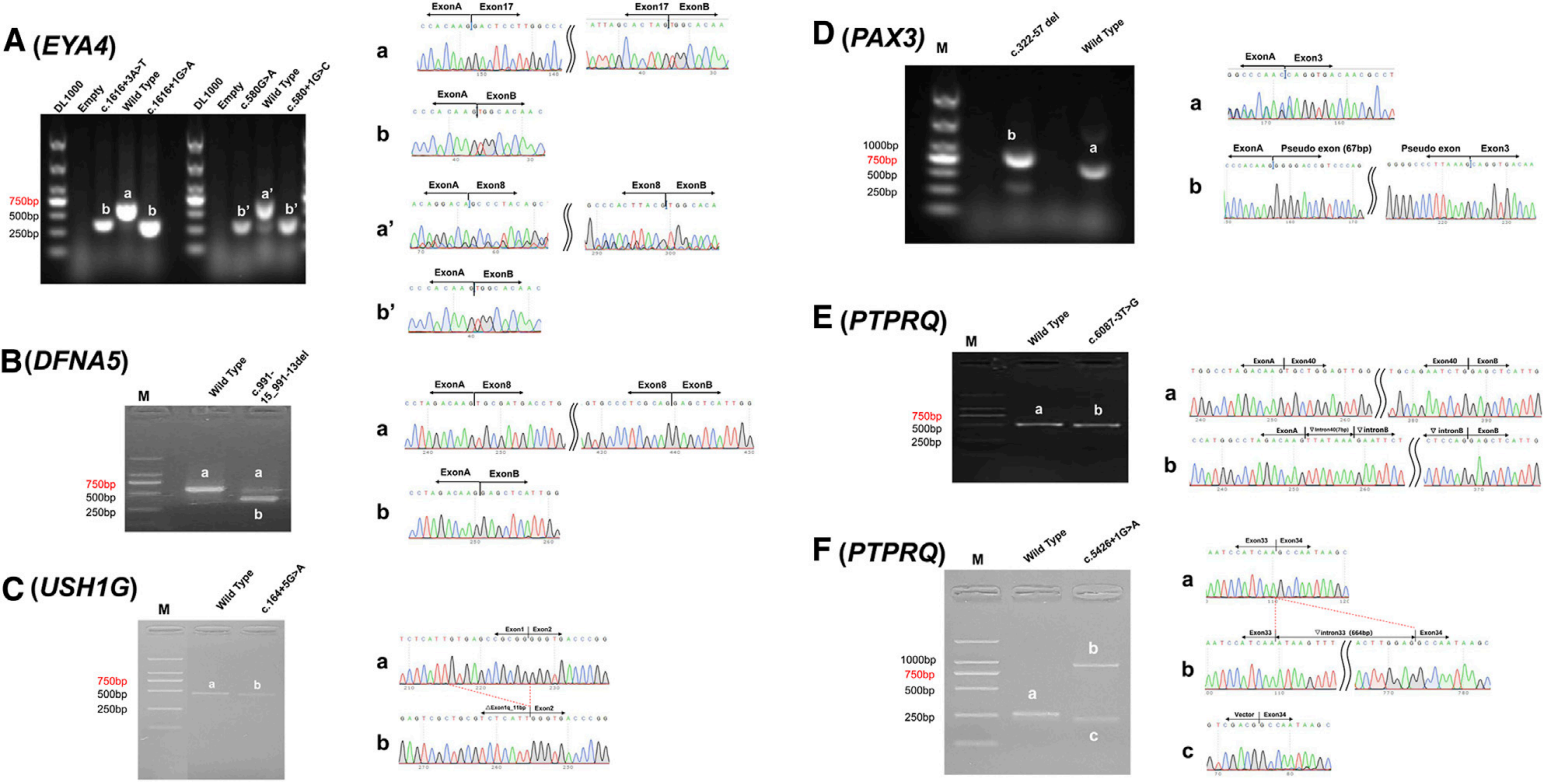
Minigene assay analysis of c.1616+3A > T and c.580G > A in *EYA4* **(A)** *,* c.991-15_991-13del in *DFNA5* **(B)**, c.164+5G > A in *USH1G* **(C)**, c.322-57_322-8del in *PAX3* **(D)**, c.6087-3T > G **(E)**, and c.5426+1G > A **(F)** in *PTPRQ.* left column. Reversed transcriptional analysis showing distinct bands (a, b, a′ and b′); right column. Sequencing results of the corresponding bands.

For all pathogenic variants identified in this study, the associated genes have extremely low expression in peripheral blood (Table 2), preventing *in vivo* analysis directly using samples from the patients. By *in vitro* minigene assay, the c.1616+3A > T and c.580G > A variants in *EYA4* and the c.991-15_991-13del variant in *DFNA5* generated a shorter spliced product missing the entire exon according to the sequencing results (Figures 3A,B). These three variants probably disrupt their original splicing site and lead to exon skipping. The rest four candidate variants generated spliced products with more complicated pattern, including exon shortening of 11bp from the 3′ end for c.164+5G > A in *USH1G* (Figure 3C), intron retention of 67 bp from the 5′ end for c.322-57_322-8del in *PAX3* (Figure 3D), exon skipping and partial intron retention for both c.5426+1G > A and c.6087-3T > G variants in *PTPRQ* (Figures 3E,F). These four variants probably disrupt the original splice sites and activate alternative ones in the exon or intron.

## Discussion

Targeted or whole-exome NGS has been increasingly employed for mutation screening of heterogeneous diseases such as deafness. The routine sequencing data analysis usually focus on non-synonymous variants in exon and CSS variant only, while the potential NCSS variants are often ignored. Though the exact prevalence of the NCSS pathogenic variants is not clear for hereditary hearing loss, several previous reports and our current study has suggested that it very likely be notable, especially in familial cases with no pathogenic variants identified in known deafness genes (Booth et al., 2018). In this paper, we propose a workflow for detection and verification of NCSS mutations by NGS (Supplementary Figure S1). The modified filtering criteria evaluate all variants, intronic or exonic, synonymous or non-synonymous, for potential negative effect on splicing, which will be followed by bioinformatic analysis and functional verification by either reversed transcriptional PCR or minigene analysis. Other helpful criteria include intrafamilial phenotypic co-segregation, consistency with previously reported gene-specific genotype-phenotype correlation, and identification of pathogenic mutations in both alleles of the recessive causative genes.

In this study, multiplex, autosomal dominant families A-D have pedigrees large enough for effective intrafamilial phenotypic co-segregation analysis. The compound heterozygous (*PTPRQ*) or homozygous (*USH1G*) mutations in Family E and F are in agreement with the presumable recessive inheritance as reported by numerous previously studies (Sang et al., 2015; Wu et al., 2018; D'Esposito et al., 2021). Affected members within each family have audiograms that show similar hearing phenotype and are consistent with previous reports for genotype-phenotype correlation of *EYA4*, *DFNA5*, *PAX3*, *PTPRQ* and *USH1G*. Family D has additional features perfectly matched to WS3, for which *PAX3* is the main causative gene. With these supporting evidences, we were able to narrow down a list of candidate NCSS variants including several that were originally missed by routine NGS filtering process.

In this paper, we predicted the pathogenicity of the six NCSS variants by three computational prediction software HSF, NNSPLICE and NetGene2 (Table 2). Though all six variants were predicted to affect the RNA splicing by at least one computational tool, it remains to be functionally verified as prediction tools alone frequently generate false positive or false negative results. In this aspect, the simplest and most effective approach is targeted sequences reversed transcriptional PCR in tissues of the lesion (Valero et al., 2019). This approach, however, rarely works for deafness-causative genes, as the cochlear tissue is difficult to obtain and none of five genes involved in this study are expressed in the peripheral blood. In this case, minigene analysis provides an *in vitro* assay for testing RNA splicing effect of NCSS variants, which can be used to confirm whether potential pathogenic variants affect splicing efficiency or activate variable hidden splicing sites (Booth et al., 2018). It also remains to be verified by other hearing-impaired patients carrying the same NCSS variants, which will provide further genetic evidence in support for its pathogenicity.

Based on the minigene analysis results, we concluded that splice site variants at the classical (type I) and junction (type II) region often directly destroy the original acceptor or donor splice site, leading to exon skipping, such as the c.1616+3A > T and c.580G > A variants in *EYA4* (Figure 3A). Sometimes NCSS variants can weaken the recognition of the donor or acceptor splicing site and activate a cryptic splicing site in the intron, resulting in exon skipping and/or partial intron retention, such as the c.5426+1G > A and c.6087-3T > G variants in *PTPRQ* (Figure 3F), or activate a cryptic splicing site in the exon, resulting in shortening of the exon, such as the c.164+5G > A variant in *USH1G* (Figure 3C). Mutations in the deep intron region (type IV) can destroy the branch point with polypyrimidine tract, resulting in exon skipping, such as c.991-15_991-13del in *DFNA5* (Figure 3B), or activate a cryptic splicing site in the deep intron, resulting in partial intron retention, such as the c.322-57_322-8del variant in *PAX3* (Figure 3D).

The c.580G > A variant in *EYA4* is of particular interest, as under most circumstance it will be interpreted as a p. Asp194Asn missense mutation at a very conservative amino acid position. Though most of the reported *EYA4* mutations are truncating mutations leading to haploinsufficiency (Zhang et al., 2004), at least four *EYA4* missense mutations have been reported to lead to non-syndromic deafness DFNA10(Tan et al., 2014; Liu et al., 2015; Sun et al., 2015; Cesca et al., 2018). However, our results showed that the c.580C > G variant disrupts the pre-mRNA splicing of *EYA4*, resulting in exon8 (143 bases) skipping and presumably early termination of the protein translation. In light of this discovery, we designed minigene experiment to analyze the other four previously reported *EYA4* missense mutations c.511G > C, c.978C > G, c.1301T > A and c.1643C > G, which all tested negative for any splicing abnormality (Supplementary Figure S2). Nevertheless, it is worth noting that exonic synonymous variants or benign amino acid substitutions may be hidden NCSS variants such as the c.580C > G variant in *EYA4*, which may deserve re-evaluation and in-depth study.

In conclusion, we identified and functionally verified 6 NCSS variants in *EYA4*, *PAX3*, *PTPRQ*, and *USH1G* in Chinese Han families with sensorineural deafness. The NCSS variants may be an important cause of genetic hearing loss that demands closer attention in genetic diagnosis.

## Data Availability

The original contributions presented in the study are publicly available. This data can be found here: PRJNA779459.
