# Supplementary material for: Detection and Functional Verification of Noncanonical Splice Site Mutations in Hereditary Deafness
Source: Front Genet. 2021 Dec 8;12:773922. doi: 10.3389/fgene.2021.773922 (PMC8693380; doi:10.3389/fgene.2021.773922)
Supplement: Supplementary file 1 [file DataSheet1.docx]

Supplementary Material

## Supplementary Table 1. List of the 144 targeted deafness genes for NGS.

| [*ACTG1*](http://www.ncbi.nlm.nih.gov/omim/102560)*^D^* | [*COL9A1*](http://www.ncbi.nlm.nih.gov/omim/120210)*^R^* | *FGF8 ^D^* | [*HSD17B4*](http://omim.org/entry/601860) *^R^* | [*MYO15A*](http://www.ncbi.nlm.nih.gov/omim/602666) *^R^* | *PROKR2^D/R^* | [*TBC1D24*](http://www.omim.org/entry/613577) *^D/R^* |
| --- | --- | --- | --- | --- | --- | --- |
| *ALX3^R^* | [*COL9A2*](http://omim.org/entry/120260) *^D/R^* | *FGFR1 ^D^* | *IL13^D^* | [*MYO1A*](http://www.ncbi.nlm.nih.gov/omim/601478) *^D^* | [*PRPS1*](http://www.ncbi.nlm.nih.gov/omim/311850)*^XLR^* | [*TCOF1*](http://www.ncbi.nlm.nih.gov/omim/606847) *^D^* |
| [*BSND*](http://www.ncbi.nlm.nih.gov/omim/606412) *^R^* | *COMT2 ^R^* | *FGFR3 ^D^* | [*ILDR1*](http://www.ncbi.nlm.nih.gov/omim/609739) *^R^* | [*MYO3A*](http://www.ncbi.nlm.nih.gov/omim/606808) *^R^* | [*PTPRQ*](http://omim.org/entry/603317) *^R^* | [*TECTA*](http://www.ncbi.nlm.nih.gov/omim/602574) *^D/R^* |
| *CABP2 ^R^* | [*CRYM*](http://www.ncbi.nlm.nih.gov/omim/123740) *^D^* | *FLNA^XLR^* | [*KARS*](http://omim.org/entry/601421) *^R^* | [*MYO6*](http://www.ncbi.nlm.nih.gov/omim/600970) *^D/R^* | [*RDX*](http://www.ncbi.nlm.nih.gov/omim/179410) *^R^* | *TIMM8A ^XLR^* |
| [*CCDC50*](http://www.ncbi.nlm.nih.gov/omim/611051) *^D^* | [*DFNA5*](http://www.ncbi.nlm.nih.gov/omim/600994) *^D^* | [*FOXI1*](http://www.ncbi.nlm.nih.gov/omim/601093) *^R^* | [*KCNE1*](http://www.ncbi.nlm.nih.gov/omim/176261) *^R^* | [*MYO7A*](http://www.ncbi.nlm.nih.gov/omim/276903) *^D/R^* | *RPGR ^XLD^* | [*TJP2*](http://www.ncbi.nlm.nih.gov/omim/607709) *^R^* |
| [*CDH23*](http://www.ncbi.nlm.nih.gov/omim/605516)*^R^* | *DFNB31 ^R^* | *FREM1 ^R^* | [*KCNJ10*](http://omim.org/entry/602208) *^R^* | [*NDP*](http://www.ncbi.nlm.nih.gov/omim/300658) *^XLR^* | *SALL1 ^D^* | [*TMC1*](http://www.ncbi.nlm.nih.gov/omim/606706) *^D/R^* |
| [*CEACAM16*](http://www.ncbi.nlm.nih.gov/pubmed/21368133) *^D^* | *DFNB59 ^R^* | *GATA3 ^D^* | [*KCNQ1*](http://www.ncbi.nlm.nih.gov/omim/607542) *^D^* | *NF2 ^D^* | *SALL4 ^D^* | [*TMIE*](http://www.ncbi.nlm.nih.gov/omim/607237) *^R^* |
| *CHD7^D^* | *DIABLO ^D^* | *GIPC3 ^R^* | [*KCNQ4*](http://www.ncbi.nlm.nih.gov/omim/603537) *^D^* | [*OTOA*](http://www.ncbi.nlm.nih.gov/omim/607038) *^R^* | [*SANS*](http://www.ncbi.nlm.nih.gov/omim/607696) *^R^* | [*TMPRSS3*](http://www.ncbi.nlm.nih.gov/omim/605511) *^R^* |
| [*CIB2*](http://omim.org/entry/605564) *^R^* | [*DIAPH1*](http://www.ncbi.nlm.nih.gov/omim/602121) *^D^* | [*GJB2*](http://www.ncbi.nlm.nih.gov/omim/121011) *^D/R^* | *KRT9 ^D^* | [*OTOF*](http://www.ncbi.nlm.nih.gov/omim/603681) *^R^* | *SEC23A ^R^* | [*TNC*](http://www.omim.org/entry/187380) *^D^* |
| [*CLDN14*](http://www.ncbi.nlm.nih.gov/omim/605608) *^R^* | *DIAPH3 ^D^* | *GJB3 ^D/R^* | *LAMA3 ^R^* | [*P2RX2*](http://www.omim.org/entry/600844) *^D^* | [*SEMA3E*](http://www.omim.org/entry/608166) *^D^* | [*TPRN*](http://www.ncbi.nlm.nih.gov/omim/613354) *^R^* |
| *CLPP ^R^* | [*DSPP*](http://www.ncbi.nlm.nih.gov/omim/125485) *^D^* | [*GJB6*](http://www.ncbi.nlm.nih.gov/omim/604418) *^D/R^* | [*LARS2*](http://omim.org/entry/604544) *^R^* | *PABPN1 ^D^* | [*SERPINB6*](http://omim.org/entry/173321) *^R^* | [*TRIOBP*](http://www.ncbi.nlm.nih.gov/omim/609761) *^R^* |
| [*CLRN1*](http://www.ncbi.nlm.nih.gov/omim/606397) *^R^* | *ECM1 ^R^* | *GPR98 ^R^* | [*LHFPL5*](http://www.ncbi.nlm.nih.gov/omim/609427) *^D^* | [*PAX3*](http://www.ncbi.nlm.nih.gov/omim/606597) *^D/R^* | [*SIX1*](http://www.omim.org/entry/601205) *^D^* | *TRMU ^R^* |
| [*COCH*](http://www.ncbi.nlm.nih.gov/omim/603196) *^D^* | [*EDN3*](http://www.ncbi.nlm.nih.gov/omim/131242)*^D/R^* | [*GPSM2*](http://omim.org/entry/609245) *^R^* | [*LOXHD1*](http://www.ncbi.nlm.nih.gov/omim/613072) *^R^* | [*PCDH15*](http://www.ncbi.nlm.nih.gov/omim/605514) *^R^* | [*SIX5*](http://www.ncbi.nlm.nih.gov/omim/600963) *^D^* | [*TSPEAR*](http://www.omim.org/entry/612920) *^R^* |
| [*COL11A1*](http://www.ncbi.nlm.nih.gov/omim/120280) *^D/R^* | [*EDNRB*](http://www.ncbi.nlm.nih.gov/omim/131244) *^D/R^* | *GRHL2 ^D^* | *LRTOMT ^R^* | [*PDZD7*](http://www.ncbi.nlm.nih.gov/omim/612971) *^R^* | [*SLC17A8*](http://omim.org/entry/607557) *^D^* | [*USH1C*](http://www.ncbi.nlm.nih.gov/omim/605242) *^R^* |
| [*COL11A2*](http://www.ncbi.nlm.nih.gov/omim/120290) *^D/R^* | [*ELMOD3*](http://www.omim.org/entry/615427) *^R^* | [*GRXCR1*](http://www.ncbi.nlm.nih.gov/omim/613283) *^R^* | [*MARVELD2*](http://www.ncbi.nlm.nih.gov/omim/610572) *^R^* | [*PNPT1*](http://omim.org/entry/610316?search=PNPT1&highlight=pnpt1) *^R^* | [*SLC26A4*](http://www.ncbi.nlm.nih.gov/omim/605646) *^R^* | *USH1G ^R^* |
| [*COL2A1*](http://www.ncbi.nlm.nih.gov/omim/120140) *^D^* | [*ESPN*](http://www.ncbi.nlm.nih.gov/omim/606351) *^R^* | *HARS ^R^* | *MIR96 ^D^* | [*POLR1C*](http://www.omim.org/entry/610060) *^R^* | [*SLC26A5*](http://www.ncbi.nlm.nih.gov/omim/604943) *^R^* | [*USH2A*](http://www.ncbi.nlm.nih.gov/omim/276901) *^R^* |
| [*COL4A3*](http://www.ncbi.nlm.nih.gov/omim/120070)*^D/R^* | [*ESRRB*](http://www.ncbi.nlm.nih.gov/omim/602167) *^R^* | [*HARS2*](http://omim.org/entry/600783) *^R^* | [*MITF*](http://www.ncbi.nlm.nih.gov/omim/156845) *^D^* | [*POLR1D*](http://www.omim.org/entry/613715) *^D^* | [*SMPX*](http://omim.org/entry/300226)*^XLD^* | [*WFS1*](http://www.ncbi.nlm.nih.gov/omim/606201) *^D^* |
| [*COL4A4*](http://www.ncbi.nlm.nih.gov/omim/120131) *^R^* | [*EYA1*](http://www.ncbi.nlm.nih.gov/omim/601653) *^D^* | [*HGF*](http://www.ncbi.nlm.nih.gov/omim/142409) *^R^* | [*MSRB3*](http://www.ncbi.nlm.nih.gov/omim/613719) *^R^* | [*POU3F4*](http://www.ncbi.nlm.nih.gov/omim/300039) *^XLR^* | [*SNAI2*](http://www.ncbi.nlm.nih.gov/omim/602150) *^R^* | *WHRN ^R^* |
| [*COL4A5*](http://www.ncbi.nlm.nih.gov/omim/303630) *^XLR^* | [*EYA4*](http://www.ncbi.nlm.nih.gov/omim/603550) *^D^* | *HMX1 ^R^* | [*MYH14*](http://www.ncbi.nlm.nih.gov/omim/608568) *^D^* | [*POU4F3*](http://www.ncbi.nlm.nih.gov/omim/602460) *^D^* | [*SOX10*](http://www.ncbi.nlm.nih.gov/omim/602229) *^D^* | *NELL2^U^* |
| [*COL4A6*](http://www.omim.org/entry/303631) *^XLR^* | *FGF3 ^R^* | *HOXA2 ^D/R^* | [*MYH9*](http://www.ncbi.nlm.nih.gov/omim/160775) *^D^* | *PROK2 ^D^* | [*STRC*](http://www.ncbi.nlm.nih.gov/omim/606440) *^R^* | *KITLG ^D^* |
| *COL1A1 ^D^* | *COL1A2^D^* | *OTOG ^R^* | *OTOGL ^R^* |  |  |  |

D: autosomal dominant, n=48; R: autosomal recessive, n=69; D/R: autosomal dominant or recessive, n=17; XLD: X-linked dominant, n=2; XLR: X-linked recessive, n=7

## Supplementary Table 2. Sequence of 8 minigenes.

| EYA4-17-WT:  CTCGAGATGATCTATTCCAGACAGAAGGGAATCTAAACTAGATATTCTGAGACAATATTCATCTCTCGACTCTGCCTTGGTTTTTTGGTGTTGCAGGACTCCTTGGCCCTGCCAAGAGGGATGCCTGGCTACAGTTAAGGGCAGAGATTGAAGGTCTGACAGATTCCTGGCTAACAAATGCACTTAAGTCTTTATCAATTATTAGCACTAGGTAAGTGGAATTGTTACCTTTCTATGTGTATCGTATTGAAGATTTAGTTCTAATTGTACAACATAGAAAAAATGTTTTAATTCCTGGATCC |
| --- |
| EYA4-17-MT:  CTCGAGATGATCTATTCCAGACAGAAGGGAATCTAAACTAGATATTCTGAGACAATATTCATCTCTCGACTCTGCCTTGGTTTTTTGGTGTTGCAGGACTCCTTGGCCCTGCCAAGAGGGATGCCTGGCTACAGTTAAGGGCAGAGATTGAAGGTCTGACAGATTCCTGGCTAACAAATGCACTTAAGTCTTTATCAATTATTAGCACTAGGTTAGTGGAATTGTTACCTTTCTATGTGTATCGTATTGAAGATTTAGTTCTAATTGTACAACATAGAAAAAATGTTTTAATTCCTGGATCC |
| EYA4-17-MTCTL:  CTCGAGATGATCTATTCCAGACAGAAGGGAATCTAAACTAGATATTCTGAGACAATATTCATCTCTCGACTCTGCCTTGGTTTTTTGGTGTTGCAGGACTCCTTGGCCCTGCCAAGAGGGATGCCTGGCTACAGTTAAGGGCAGAGATTGAAGGTCTGACAGATTCCTGGCTAACAAATGCACTTAAGTCTTTATCAATTATTAGCACTAGATAAGTGGAATTGTTACCTTTCTATGTGTATCGTATTGAAGATTTAGTTCTAATTGTACAACATAGAAAAAATGTTTTAATTCCTGGATCC |
| EYA4-8-WT:  CTCGAGGATTACTTCCTGGATATTATAGGGTTTCACTGAATCTAGGCTGTCTAAATTAATACACAGTCTTTGTTGCCACAGTAATGCTATTTTTCTGATATTTAGGCCCTATCCACACATTCTTTCTACACCAGCAGCTCAAACAATGTCTGCCTATGCAGGCCAGACTCAGTATTCGGGGATGCAGCAGCCAGCCGTCTACACAGCCTACTCACAGACAGGACAGCCCTACAGCTTGCCCACTTACGGTATTTCACATCTTCTGTTTTCTTCTTTGGTTATAGGCAGGTAATCCTGCTGGCTGGTAGCTTTGTATTCTATTGTAGGTTACAACTTAGAAGGAAAATGGATCC |
| EYA4-8-MT:  TCGAGGATTACTTCCTGGATATTATAGGGTTTCACTGAATCTAGGCTGTCTAAATTAATACACAGTCTTTGTTGCCACAGTAATGCTATTTTTCTGATATTTAGGCCCTATCCACACATTCTTTCTACACCAGCAGCTCAAACAATGTCTGCCTATGCAGGCCAGACTCAGTATTCGGGGATGCAGCAGCCAGCCGTCTACACAGCCTACTCACAGACAGGACAGCCCTACAGCTTGCCCACTTACAGTATTTCACATCTTCTGTTTTCTTCTTTGGTTATAGGCAGGTAATCCTGCTGGCTGGTAGCTTTGTATTCTATTGTAGGTTACAACTTAGAAGGAAAATGGATCC |
| EYA4-8-MTCTL:  TCGAGGATTACTTCCTGGATATTATAGGGTTTCACTGAATCTAGGCTGTCTAAATTAATACACAGTCTTTGTTGCCACAGTAATGCTATTTTTCTGATATTTAGGCCCTATCCACACATTCTTTCTACACCAGCAGCTCAAACAATGTCTGCCTATGCAGGCCAGACTCAGTATTCGGGGATGCAGCAGCCAGCCGTCTACACAGCCTACTCACAGACAGGACAGCCCTACAGCTTGCCCACTTACGCTATTTCACATCTTCTGTTTTCTTCTTTGGTTATAGGCAGGTAATCCTGCTGGCTGGTAGCTTTGTATTCTATTGTAGGTTACAACTTAGAAGGAAAATGGATCC |
| PAX3-3-WT：  CTCGAGCCTCAGAGAGTTCAGGTCTTTTTGCTCACAAAGGGGGACCGTCCCAGAGGATTCAGCGAGGAGCATCCCGACAGGCCCAGAGGCGGTGGGGCCGCCGCCACCTGGCCCAGGGTACCGGGTACCAACGCCTGCCCGCCTGTTCTCTTAAAGCAGGTGACAACGCCTGACGTGGAGAAGAAAATTGAGGAATACAAAAGAGAGAACCCGGGCATGTTCAGCTGGGAAATCCGAGACAAATTACTCAAGGACGCGGTCTGTGATCGAAACACCGTGCCGTCAGGTACTAGGCCCATTAACCTCTCCCCGAGGCGCGACAGTCAGTCGCTATTACCCCGGCACGTCGACCCAGACGTAATCACCCCAATTATCGATCTCAGCTAGGATCC |
| PAX3-3-MT：CTCGAGCCTCAGAGAGTTCAGGTCTTTTTGCTCACAAAGGGGGACCGTCCCAGAGGATTCAGCGAGGAGCATCCCGACAGGCCCAGAGGCGGTGGGGCCCTTAAAGCAGGTGACAACGCCTGACGTGGAGAAGAAAATTGAGGAATACAAAAGAGAGAACCCGGGCATGTTCAGCTGGGAAATCCGAGACAAATTACTCAAGGACGCGGTCTGTGATCGAAACACCGTGCCGTCAGGTACTAGGCCCATTAACCTCTCCCCGAGGCGCGACAGTCAGTCGCTATTACCCCGGCACGTCGACCCAGACGTAATCACCCCAATTATCGATCTCAGCTAGGATCC |

## Supplementary Table 3. Primer sequence for minigene fishing and restriction site introduction.

| **Primer name** | **Sequence（5’- -3’）** |
| --- | --- |
| DFNA5-50831-F | ctacagttttgttttttgt |
| DFNA5-51221-F | tgatcggatctggacaccaa |
| DFNA5-52390-R | atgctctccacagccccctt |
| DFNA5-52781-R | atttatcagcacatagagtt |
| pcMINI-DFNA5-BamHⅠ-F | GCTCGGATCCccccagttctgtgtgtaacc |
| pcMINI-DFNA5-EcoRⅠ-R | TGCAGAATTCgctgggactccggagtgaac |
| PTPRQ-203580-F | actttgtaatacctggtcca |
| PTPRQ-204101-F | cactcatgaaacaatactta |
| PTPRQ-205810-R | attttcttatagtttctgga |
| PTPRQ-206310-R | tcttaagaagcataaccaaa |
| pEGFP-C1-PTPRQ-KpnⅠ-MUT1-F | CGACGGTACCCGGTTACTTAGTTATAGAAAAT |
| pEGFP-C1-PTPRQ-BamHⅠ-MUT1-R | CGGTGGATCCCGAAAATTCTTCTTGAAACT |
| PTPRQ-227891-F | ctgctatgatccaggtgccc |
| PTPRQ-228280-F | ctttgagtattgatgtaatt |
| PTPRQ-229451-R | aaacctcacagttaatagtt |
| PTPRQ-229840-R | gtaacatacaaattccaatc |
| pcMINI-PTPRQ-BamHⅠ-MUT2-F | GCTCGGATCCcaacaacacaccctaaaata |
| pcMINI-PTPRQ-EcoRⅠ-MUT2-R | TGCAGAATTCtttataacttttcaaaagat |
| USH1G-F | CAGATGTCTTGGTAGTCGCG |
| USH1G-exon2-3250-R | GGAACATGTCCCGGAGCGGG |
| USH1G-intron-F | ccactctccagccttgctcacccctgggct |
| USH1G-intron-R | agcccaggggtgagcaaggctggagagtgg |
| pEGFP-C1-USH1G-HindⅢ-F | GCTCAAGCTTCCATGAACGACCAGTACCAC |
| pEGFP-C1-USH1G-BamHⅠ-R | CGGTGGATCCCGCATTCCATGTGGCCCTTC |

## Supplementary Figures

**
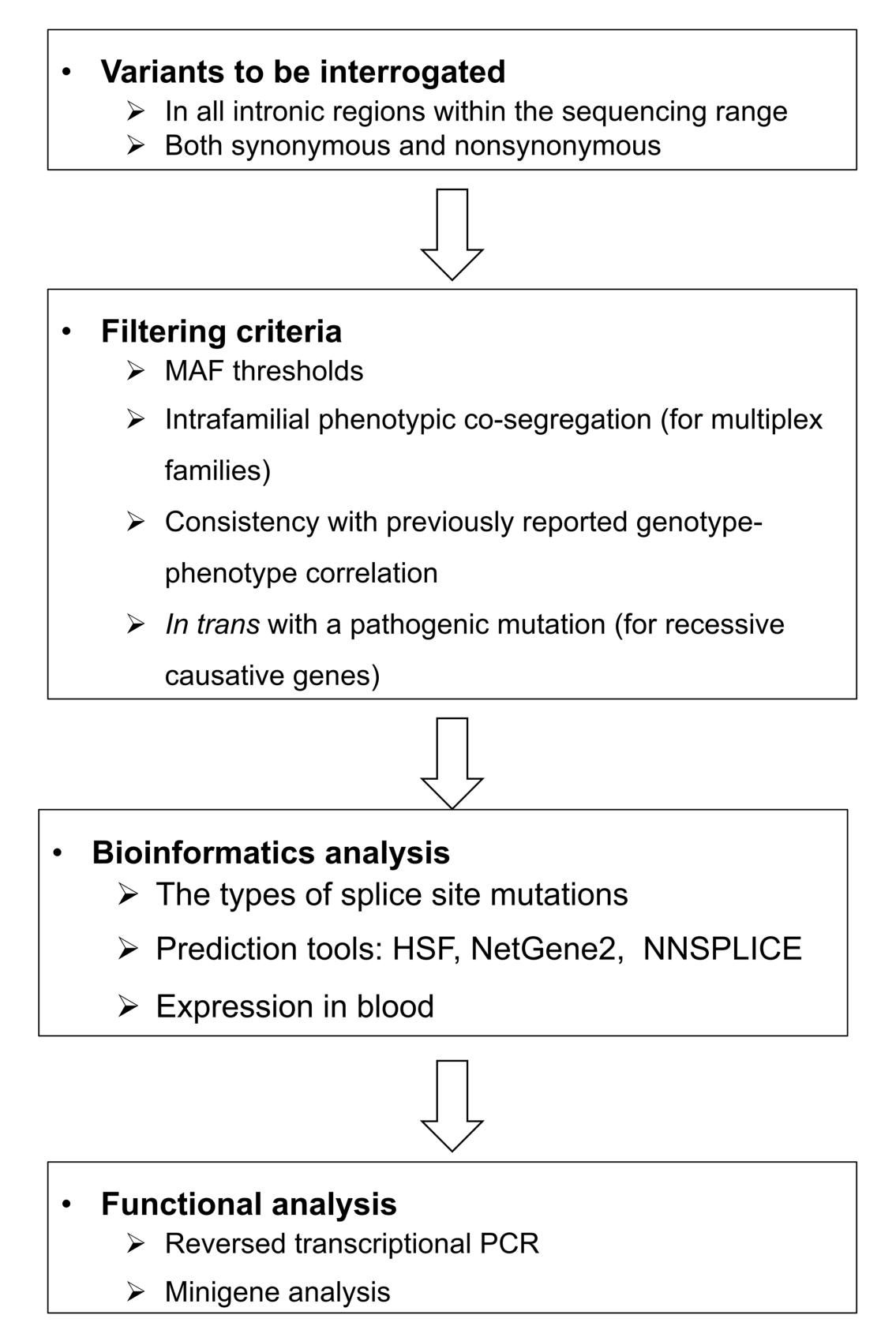
**

**Supplementary Fig.1** Schematic illustration of the workflow employed in this study for detection and verification of the NCSS variants.

**
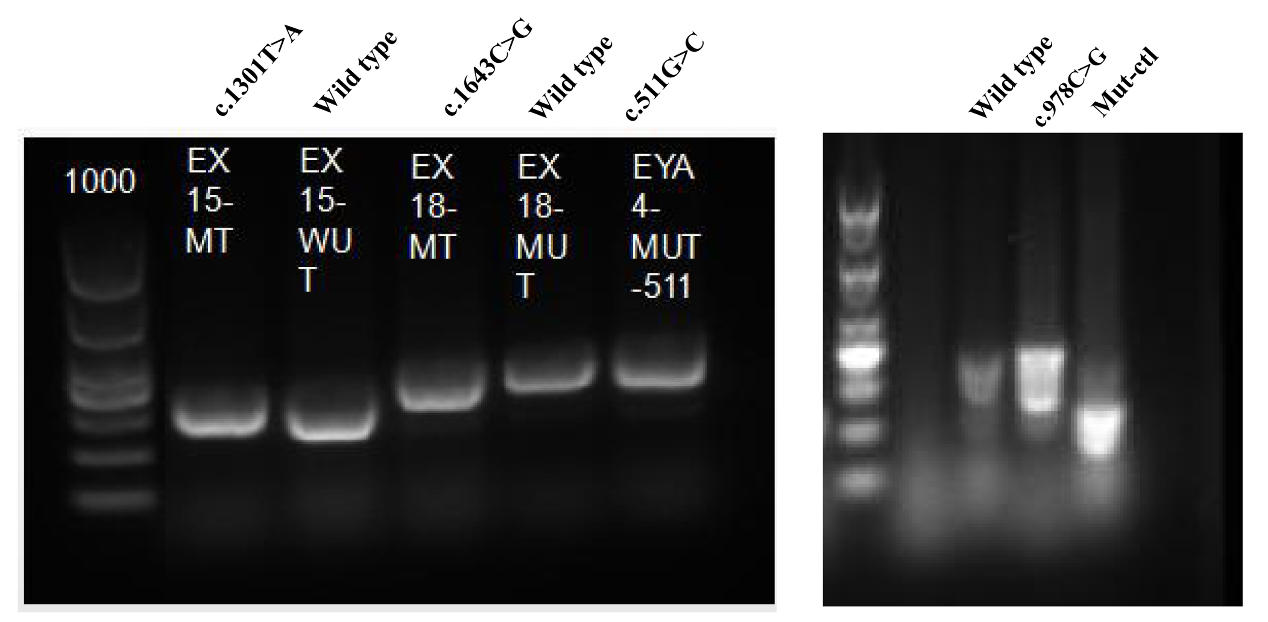
**

**Supplementary Figure 2.**  Minigene assay analysis of the c.511G>C, c.978C>G, c.1301T>A and c.1643C>G variants in *EYA4*.
